# Defective liver glycogen autophagy related to hyperinsulinemia in intrauterine growth-restricted newborn wistar rats

**DOI:** 10.1038/s41598-020-74702-9

**Published:** 2020-10-19

**Authors:** Juan de Toro-Martín, Tamara Fernández-Marcelo, Águeda González-Rodríguez, Fernando Escrivá, Ángela M. Valverde, Carmen Álvarez, Elisa Fernández-Millán

**Affiliations:** 1 Centre Nutrition, Santé et Société (NUTRISS)-Institut sur la nutrition et les aliments fonctionnels (INAF), Université Laval, Québec City, QC Canada; 2 Centro de Investigación Biomédica en Red de Diabetes y Enfermedades Metabólicas Asociadas (CIBERdem), ISCIII, 28029 Madrid, Spain; 3 Hospital Universitario Santa Cristina, Instituto de Investigación Sanitaria Princesa, Madrid, Spain; 4 Centro de Investigación Biomédica en Red de Enfermedades Hepáticas y Digestivas (CIBERehd), ISCIII, Madrid, Spain; 5 Departamento de Bioquímica y Biología Molecular, Facultad de Farmacia, UCM, Ciudad Universitaria s/n, 28040 Madrid, Spain; 6 Instituto de Investigaciones Biomédicas Alberto Sols (IIBm) (CSIC/UAM), C/ Arturo Duperier 4, 28029 Madrid, Spain

**Keywords:** Biochemistry, Diseases, Endocrinology

## Abstract

Maternal malnutrition plays a critical role in the developmental programming of later metabolic diseases susceptibility in the offspring, such as obesity and type 2 diabetes. Because the liver is the major organ that produces and supplies blood glucose, we aimed at defining the potential role of liver glycogen autophagy in the programming of glucose metabolism disturbances. To this end, newborns were obtained from pregnant Wistar rats fed ad libitum with a standard diet or 65% food-restricted during the last week of gestation. We found that newborns from undernourished mothers showed markedly high basal insulin levels whereas those of glucagon were decreased. This unbalance led to activation of the mTORC1 pathway and inhibition of hepatic autophagy compromising the adequate handling of glycogen in the very early hours of extrauterine life. Restoration of autophagy with rapamycin but not with glucagon, indicated no defect in autophagy machinery per se*,* but in signals triggered by glucagon. Taken together, these results support the notion that hyperinsulinemia is an important mechanism by which mobilization of liver glycogen by autophagy is defective in food-restricted animals. This early alteration in the hormonal control of liver glycogen autophagy may influence the risk of developing metabolic diseases later in life.

## Introduction

The perinatal period is attended by important modifications in several physiological functions and, particularly, by dramatic changes in nutrition. The interruption of the continuous transplacental fuel supply to the fetus at birth imposes an extremely rapid reprogramming of cell metabolism not only to ensure the survival during the neonatal period, but also to establish lifelong mechanisms for the adaptation to the alternating feeding and fasting of extrauterine life^1^. After delivery, and during the period of nutrient deprivation until suckling, newborn rats are sustained by the precipitous degradation of liver glycogen to produce glucose. The phosphorolytic degradation of glycogen in the cytoplasm of hepatocytes is assisted by its hydrolytic degradation into autophagic vacuoles to produce non-phosphorylated glucose^2^. Whereas phosphorolytic degradation of glycogen is mediated by the combined actions of liver glycogen phosphorylase (PYGL) and glycogen debranching enzyme, the lysosomal-derived glycogen-hydrolyzing acid α-glucosidase (GAA) is required for its lysosomal degradation by glycogen autophagy^3^. Within the first few hours of life the mechanisms of glycogenolysis and gluconeogenesis are not fully established, so for the maintenance of glucose homeostasis in the newborn it is absolutely necessary the glycogen autophagy^4,5^. This process is triggered by glucagon secreted during the hypoglycemia that follows birth, but inhibited by insulin^2,4^. However, it remains unclear how glycogen is transported to the lysosome and whether this process is somewhat different from the lysosomal degradation of glycogen observed in the adult liver.

Macroautophagy (henceforth designed ‘autophagy’) is one of the best-known types of autophagy. During autophagy, firstly the cargo components are surrounded by a double membrane to form autophagosomes that eventually fuse with the lysosome where the cargo is degraded. In nutrient-rich conditions, activation of the mammalian target of rapamycin complex 1 (mTORC1) pathway can inhibit autophagy by interacting with and phosphorylating the unc51-like kinase 1/2 complex (ULK1/2)^6^. Under fasting conditions, glucagon binding to its receptor represses mTORC1 signaling by activating AMP-activated protein kinase (AMPK)^2,7,8^. The activation of AMPK is mediated in part through the phosphorylation of the kinase on Thr^172^ by the liver kinase B1 (LKB1)^9^. Earlier studies showing that protein kinase A (PKA) phosphorylates LKB1^10,11^ provide a potential mechanism through which glucagon might repress signaling through mTORC1 and activate glycogen autophagy. Moreover, active AMPK causes the dissociation of ULK1/2 from mTORC1 by directly activating ULK1 through phosphorylation of Ser^317^ and Ser^777^^12^, thereby promoting the initiation of autophagy.

The importance of autophagy in glycogen metabolism was underscored by Pompe’s disease (glycogen storage disease type II), in which the GAA enzyme is mutated and glycogen overaccumulates in lysosomes and vesicular structures^13^. Moreover, newborn mice deficient for autophagy-related gene (*Atg)5* and *Atg7*, essential genes for autophagosome formation, show reduced number of autophagic vacuoles, live shorter than normal mice under non-suckling conditions and develop signs of nutrient and energy depletion^14,15^. Other studies have also linked obesity and type 2 diabetes (T2D) with defective hepatic autophagy compromising macromolecule turnover, such as glycogen and lipids, and causing insulin resistance^16^. However, whether alterations of the neonatal glycogen autophagy pathway in the liver may increase susceptibility to the development of later metabolic disturbances has not been systematically studied. To address this hypothesis, the current study used a programming model of intrauterine growth restriction (IUGR) validated in previous works^17,18^. Epidemiological and experimental studies on IUGR have shown reproducibly strong links between indices of fetal and infant growth retardation and impaired glucose tolerance, T2D, and metabolic syndrome development in adulthood^18,19^. It has been suggested that poor maternal and fetal nutrition, would be responsible of permanent metabolic changes in key organs leading to altered glucose homeostasis in adult life^20^. In this context, anabolic routes would be favored over catabolic ones. In agreement, we previously described that growth restricted offspring from dams undernourished through pregnancy and lactation showed evidences of improved energy storage when compared to offspring from well-nourished mothers. This was evidenced by increased liver glycogen content^17^ but impaired endogenous glucose production. Because the liver is the major organ that produces and supplies blood glucose, the correct maturation of the mechanisms for hepatic glucose output is essential in the control of glucose homeostasis. Therefore, in the present work we aimed at defining the potential role of liver glycogen autophagy in the programming of glucose metabolism disturbances.

## Results

### Early undernutrition induces massive hepatic glycogen accumulation at term but lack of glycogen mobilization immediately after birth

**Figure 1:**
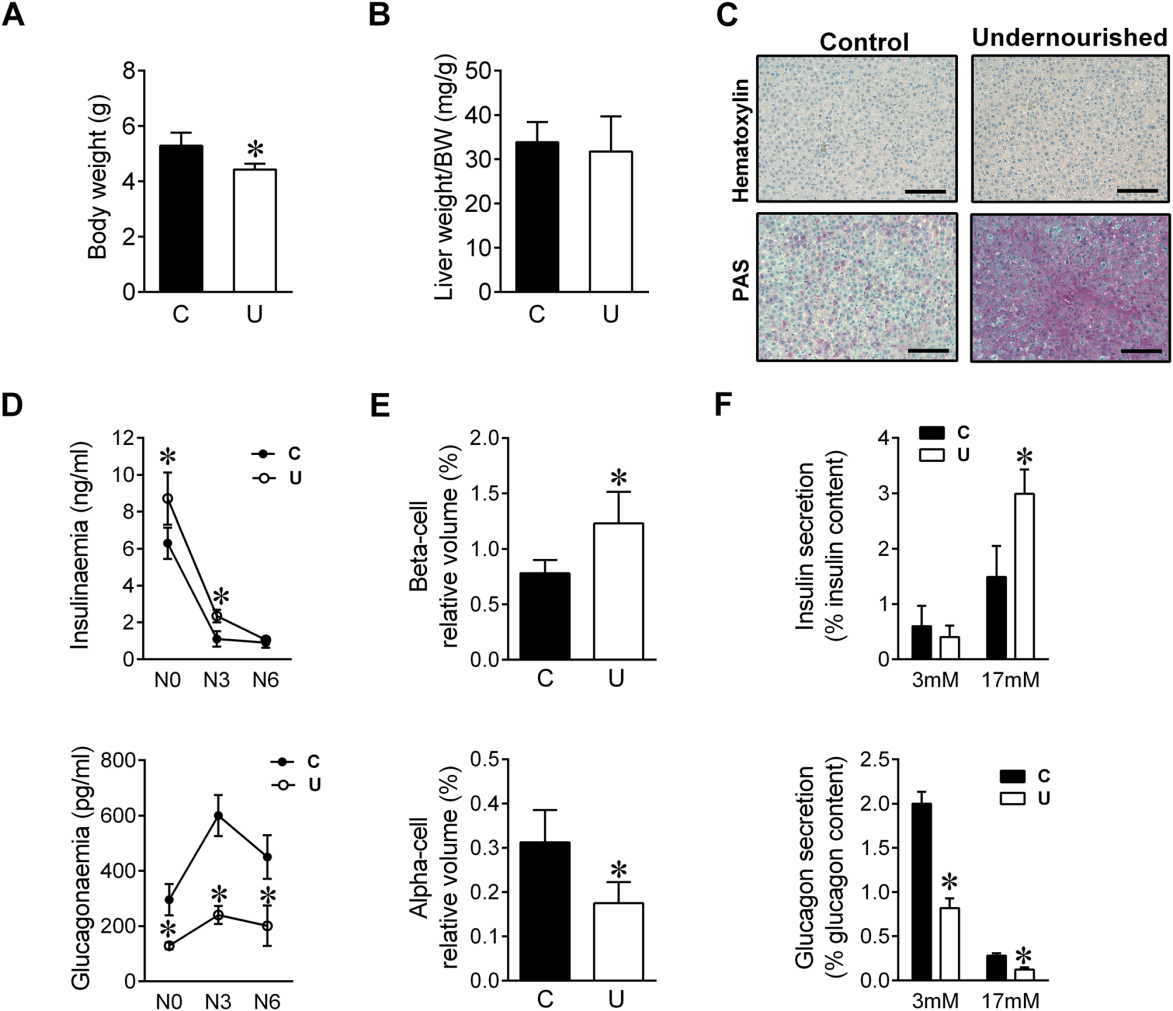
Effect of maternal food-restriction on postnatal changes in pancreatic hormone concentrations. (**A**) Body weight (BW) and (**B**) liver weight relative to BW of control (C) and undernourished (U) neonates at birth (N0), n = 14–16. (**C**) Hematoxylin and periodic acid-Schiff (PAS) staining of liver sections from C and U neonates at N0 (scale bar, 100 μm). (**D**) Postnatal changes in circulating insulin and glucagon levels of C and U neonates at birth (N0), or after 3 h (N3) or 6 h of fasting (N6), n = 6–8. (**E**) Relative beta-cell (upper graph) and alpha-cell (lower graph) volume at birth time (N0) in C and U neonates, n = 4. (**F**) Insulin (upper graph) and glucagon (lower graph) secretion in response to glucose from isolated islets of C and U neonates at N0 (n = 6–7 for each condition). All values are mean ± SD. *p < 0.05 U versus C neonates at the same time point or stimulus condition.

At birth, U neonates had significantly lower body weight (4.42 ± 0.26 g) than C animals (5.28 ± 0.56 g), (Fig. 1A). Although liver weight was also significantly lower in U neonates (132.4 ± 2.99 mg) as compared to C animals (185.5 ± 0.16 mg n = 16), no differences were found between groups when liver weight was corrected by total body weight (Fig. 1B). Moreover, periodic acid-Schiff (PAS) staining showed markedly increased glycogen storage in the liver of IUGR neonates (Fig. 1C). In agreement with other studies^1^, circulating insulin levels were very high in C animals at term, decreased dramatically after birth, and remained low during the first 6 h of life (Fig. 1D, *upper panel*). By contrast, glucagonemia raised by twofold from birth to 3 h of fasting and continued elevated after 6 h in C group (Fig. 1D, *lower panel*). Interestingly, U neonates showed significantly elevated values of serum insulin at N0 and N3 although at N6 insulinemia was similar to the C values. These results are in agreement not only with a higher pancreatic beta-cell relative mass but also with enhanced glucose-stimulated insulin secretion in islets from U newborns (Fig. 1E, F, respectively, *upper panels*). Glucagonemia was significantly lower in U neonates as compared to C animals at every time point studied, which was associated to defective alpha-cell mass and function at birth (Fig. 1D–F, respectively, *lower panels*).

**Figure 2:**
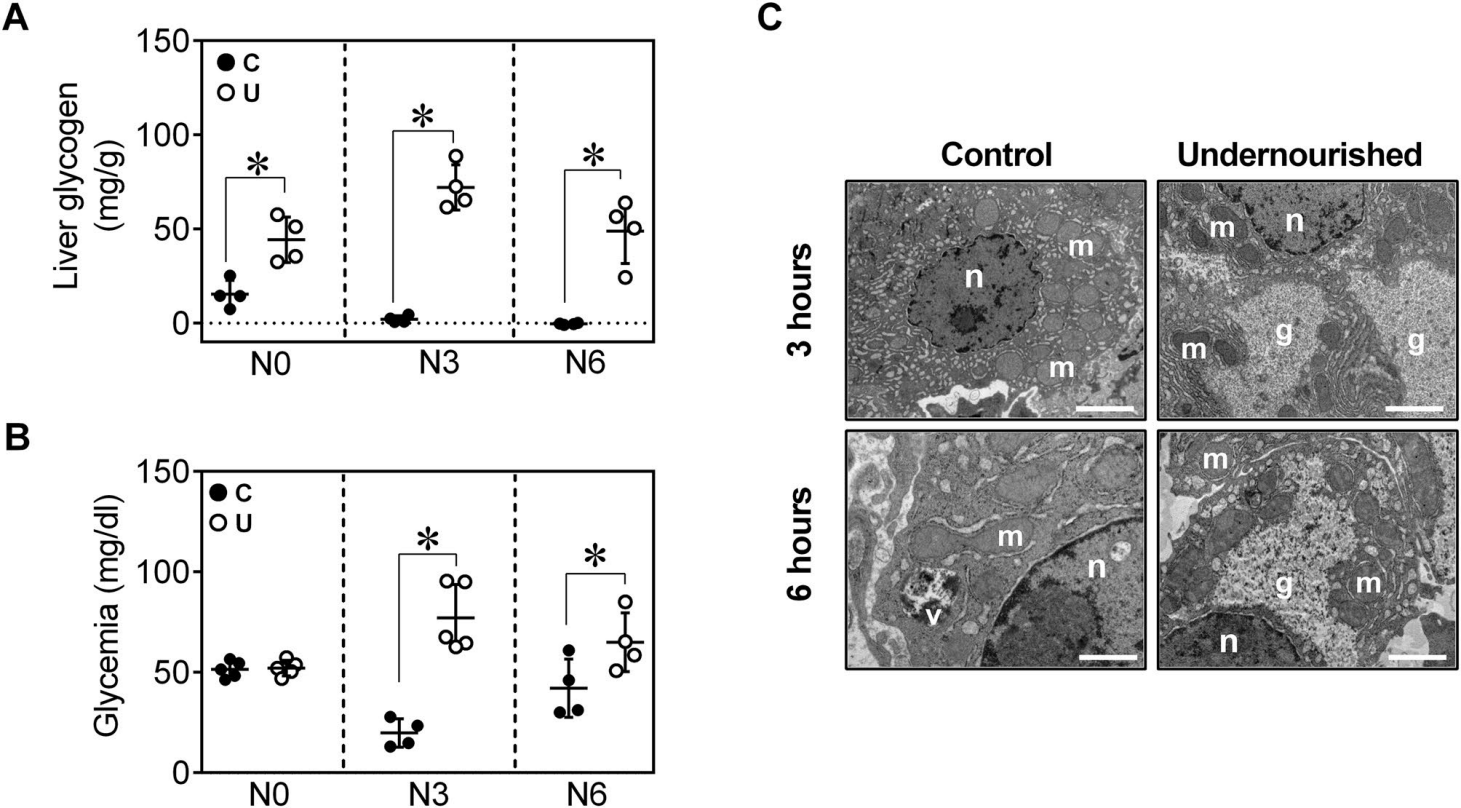
Increased hepatic glycogen storage but reduced glycogen mobilization in IUGR newborns. (**A**) Glycogen content in livers and glycemia (**B**) from C or U neonates at N0, N3 and N6, n = 4–5. (**C**) Representative electron microscopy images showing glycogen content in hepatocytes from C and U neonates at N3 and N6 (scale bar, 500 nm); n: nucleus, m: mitochondria, g: glycogen. All values are mean ± SD. *p < 0.05 U versus C neonates at the same time point.

Quantification of liver glycogen showed that U hepatocytes contained 2.7-fold higher amount of glycogen per gram of tissue than C animals at birth (Fig. 2A). The content of glycogen precipitously diminished at 3 and 6 h after birth in N3C and N6C neonates respectively, whereas glycogen stores remained significantly elevated in N3U and N6U animals (Fig. 2A, C). After 3 h of fasting, glycemia dropped in N3C neonates but it was restored in N6C to almost levels detected at birth. In sharp contrast, U neonates not only did not show a fall in blood glucose levels, but also N3U and N6U were hyperglycaemic compared to C animals (Fig. 2B). Finally, the analysis of liver sections by TEM showed vast areas of cytosolic hepatic glycogen in N3U neonates which could be still visualized after 6 h of fasting in N6U neonates, but they were almost undetectable in N6C animals (Fig. 2C).

### Increased glycogen synthesis and decreased endogenous glucose production pathways in the liver of undernourished neonates

**Figure 3:**
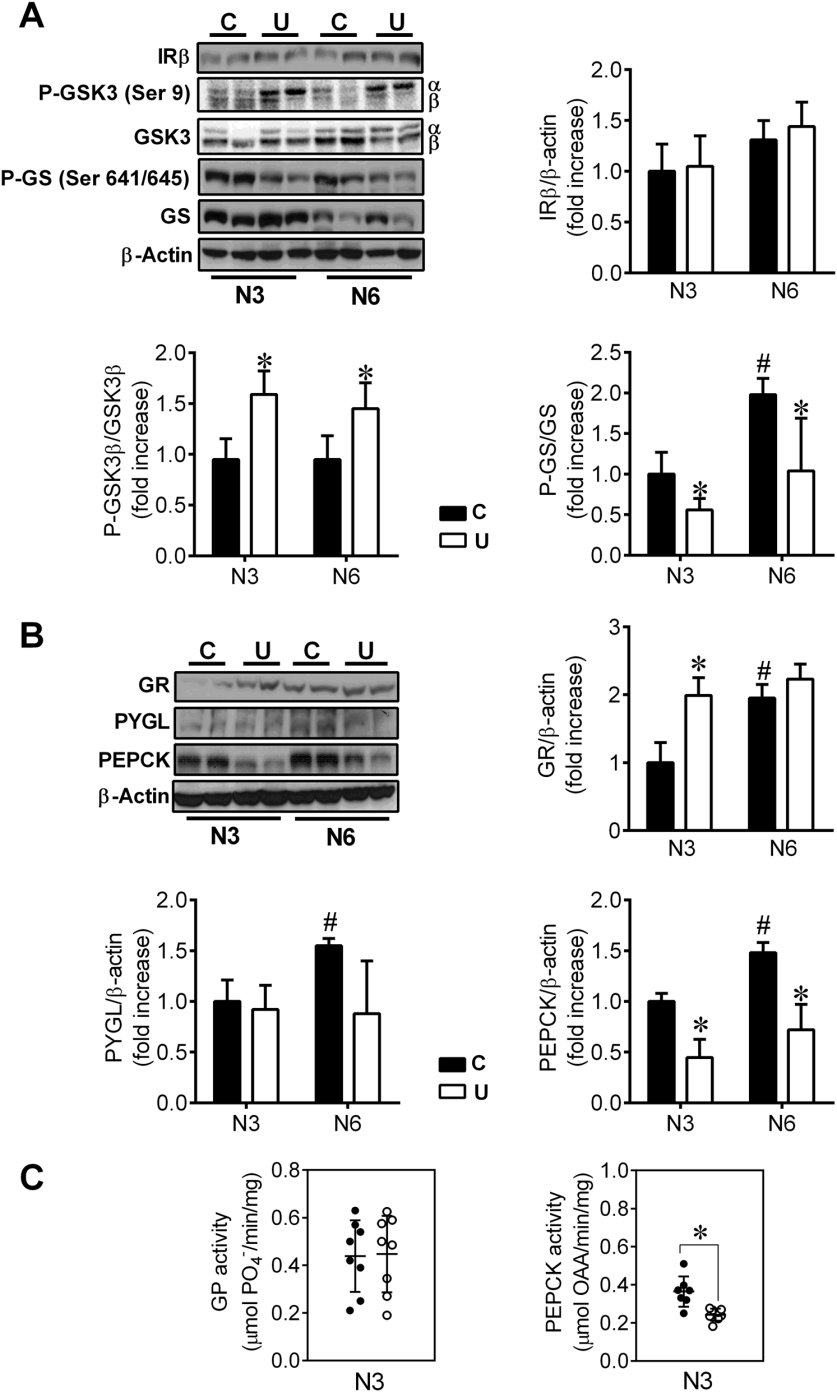
Regulation of hepatic levels of carbohydrate metabolism enzymes in response to postnatal fasting. (**A**, **B**) Activation of the pathways involved in the synthesis or degradation of glycogen was determined by immunoblotting for the indicated proteins in liver extracts from neonates after 3 or 6 h of fasting (N3 and N6, respectively). Representative blots are shown. Bars represent the densitometric quantification of all blots relative to β-actin protein levels with 6 animals per group. All values are mean ± SD. *p < 0.05 U versus C neonates at the same time point. #*p* < 0.05 N6 versus N3 within the same nutritional condition. (**C**) Enzymatic activities of the liver isoform of glycogen phosphorylase (PYGL) and phosphoenol pyruvate carboxy kinase (PEPCK) from C and U neonates at N3, n = 8. All values are mean ± SD. *p < 0.05 U versus C neonates.

Next, we analyzed the protein levels of the key enzymes of liver glycogen metabolism under fasting conditions. Regarding insulin-signalling pathway, we found no differences in the hepatic levels of insulin receptor between both C and U neonates, either at 3 or 6 h of life (Fig. 3A). However, in agreement with the glycogen accumulation detected in the liver of N3U and N6U neonates, the phosphorylated (inactive) form of the glycogen synthase kinase 3 (GSK3) was significantly increased in U animals at both time points. Concomitantly, glycogen synthase was also found more active (less phosphorylated) in U neonates (Fig. 3A). These findings are consistent with hepatic insulin hypersensitivity associated with early undernutrition, as previously reported^17^.

On the other hand, whereas the protein levels of glucagon receptor progressively increased from 3 to 6 h of life in the liver of C neonates, this increase was already evident in N3U animals (Fig. 3B). Short after birth, the liver of well-nourished newborns showed a progressive increase in the synthesis of the rate-limiting enzymes involved in endogenous glucose production, PYGL and phosphoenolpyruvate carboxykinase (PEPCK) (Fig. 3B). This event has been reported to be triggered by the concomitant rise in plasma glucagon and catecholamines and the drop in plasma insulin that occurs immediately after birth^1^.

**Figure 4:**
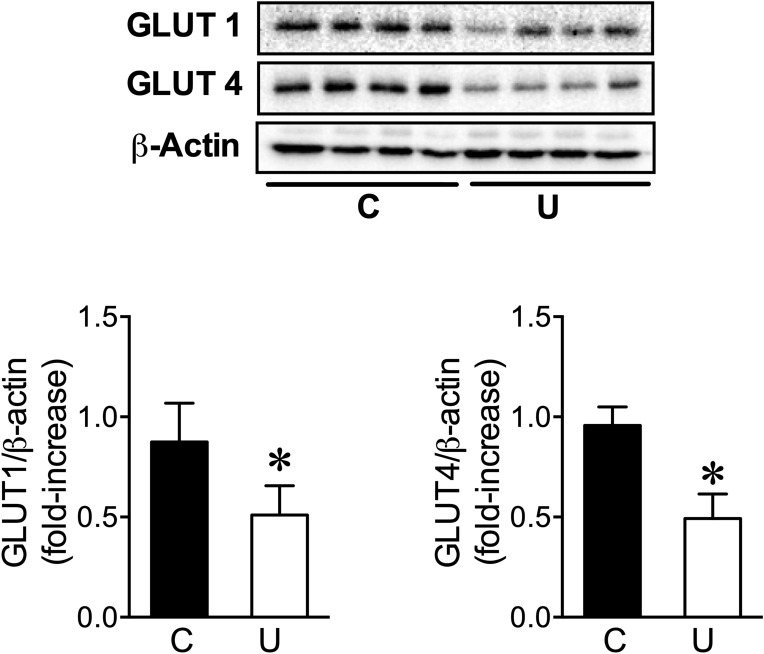
Effect of IUGR on glucose transporters content (GLUT-1 and GLUT-4) in the skeletal muscle from neonates. Top: Immunoblots for the indicated proteins in skeletal muscle homogenates from 3-h fasted neonates. Bottom: Bars represent densitometric quantification of GLUT-1 and GLUT-4 blots relative to β-actin protein levels from 6 to 8 individual animals per group. All values are mean ± SD. *p < 0.05 U versus C neonates.

In agreement with the hyperinsulinemia but the hypoglucagonemia found in U newborns, the physiological induction of PYGL and PEPCK was virtually absent in restricted animals (Fig. 3B). However, in order to explain the unexpected hyperglycemia detected in U neonates the activities of both enzymes were also analysed after 3 h of fasting. Whereas the activity of PYGL was similar in both groups of fasted animals (Fig. 3C), N3U neonates showed a 36% decrease in PEPCK activity than N3C group (Fig. 3C). Therefore, these results are not consistent with the maintenance of blood glucose levels in U newborns. Because considering tissue mass, the muscle contributes to glycemic control to a larger extent than fat, especially taking into account that newborn rats do not have fat stores at birth^21^ and because it constitutes the largest insulin-responsive tissue of the organism^22^, we then studied the muscle glucose transport system. When we analysed the effect of IUGR on skeletal muscle content of GLUT-4 and GLUT-1, we found that this condition led to a decrease in protein levels of both transporters (Fig. 4), suggesting decreased rate of glucose uptake by skeletal muscle likely contributing to the high blood glucose levels observed in U newborns.

### Hepatic autophagy flux is impaired in undernourished neonates

**Figure 5:**
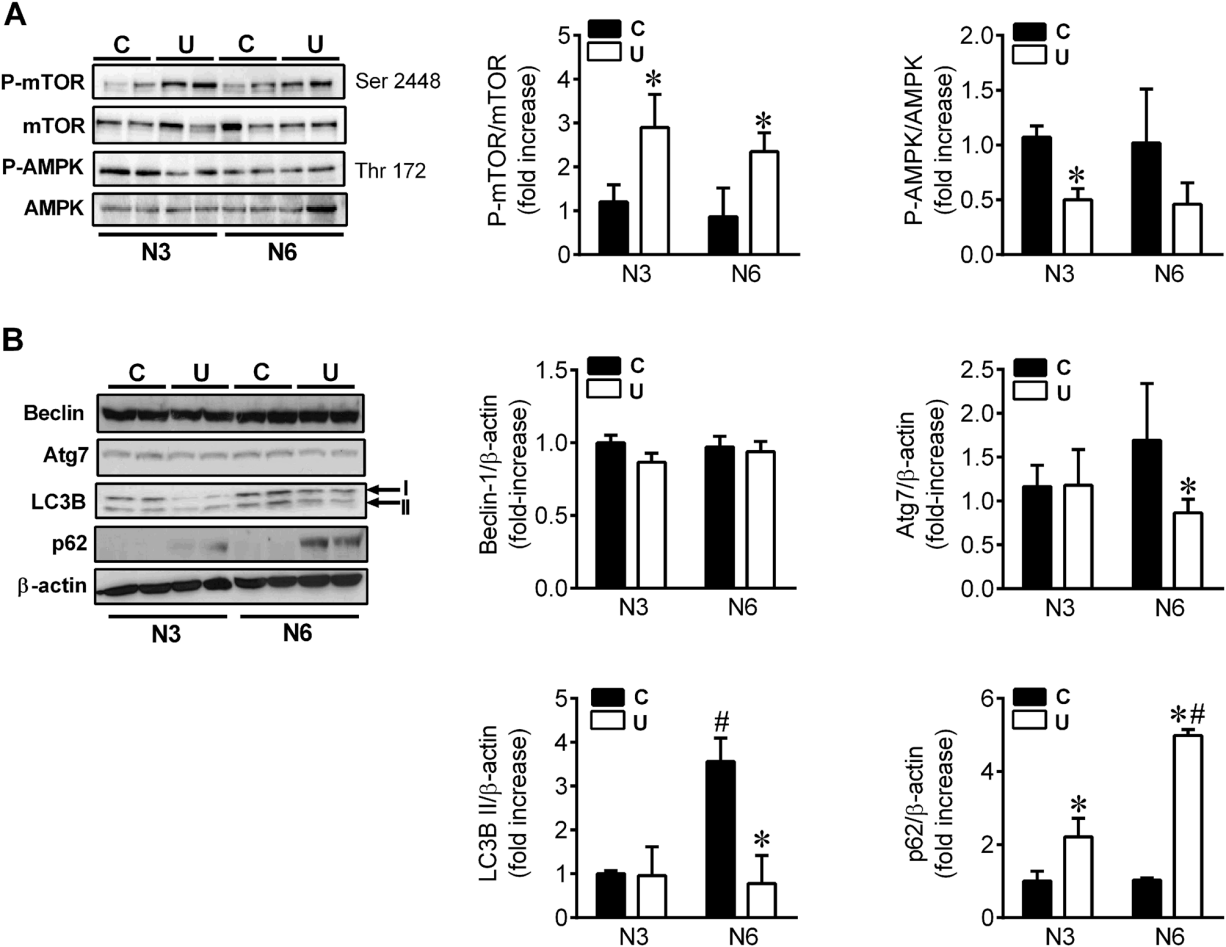
Hepatic autophagy flux is impaired in U neonates. (**A**, **B**) Immunoblots for the indicated proteins involved in autophagy induction in liver homogenates from 3-h or 6-h fasted neonates (N3 and N6, respectively). Bars represent densitometric quantification of all blots relative to β-actin protein levels from 4 to 6 individual animals per group. All values are mean ± SD. *p < 0.05 U versus C neonates at the same time point. #*p* < 0.05 N6 versus N3 within the same nutritional condition.

In conditions of nutrient deprivation, such as postnatal fasting, the binding of glucagon to its receptors in the liver leads to the activation of AMPK, as well as to the inactivation of mTORC1, promoting the induction of autophagy^7,8^. However, according to the hyperinsulinaemia showed by U neonates, together with a marked hypoglucagonemia, the activation of mTORC1 was enhanced at both 3 and 6 h of life. On the contrary, the phosphorylation of the catalytic α subunit of AMPK was significantly lower in this population compared with the C group after 3 h of fasting (Fig. 5A).

**Figure 6:**
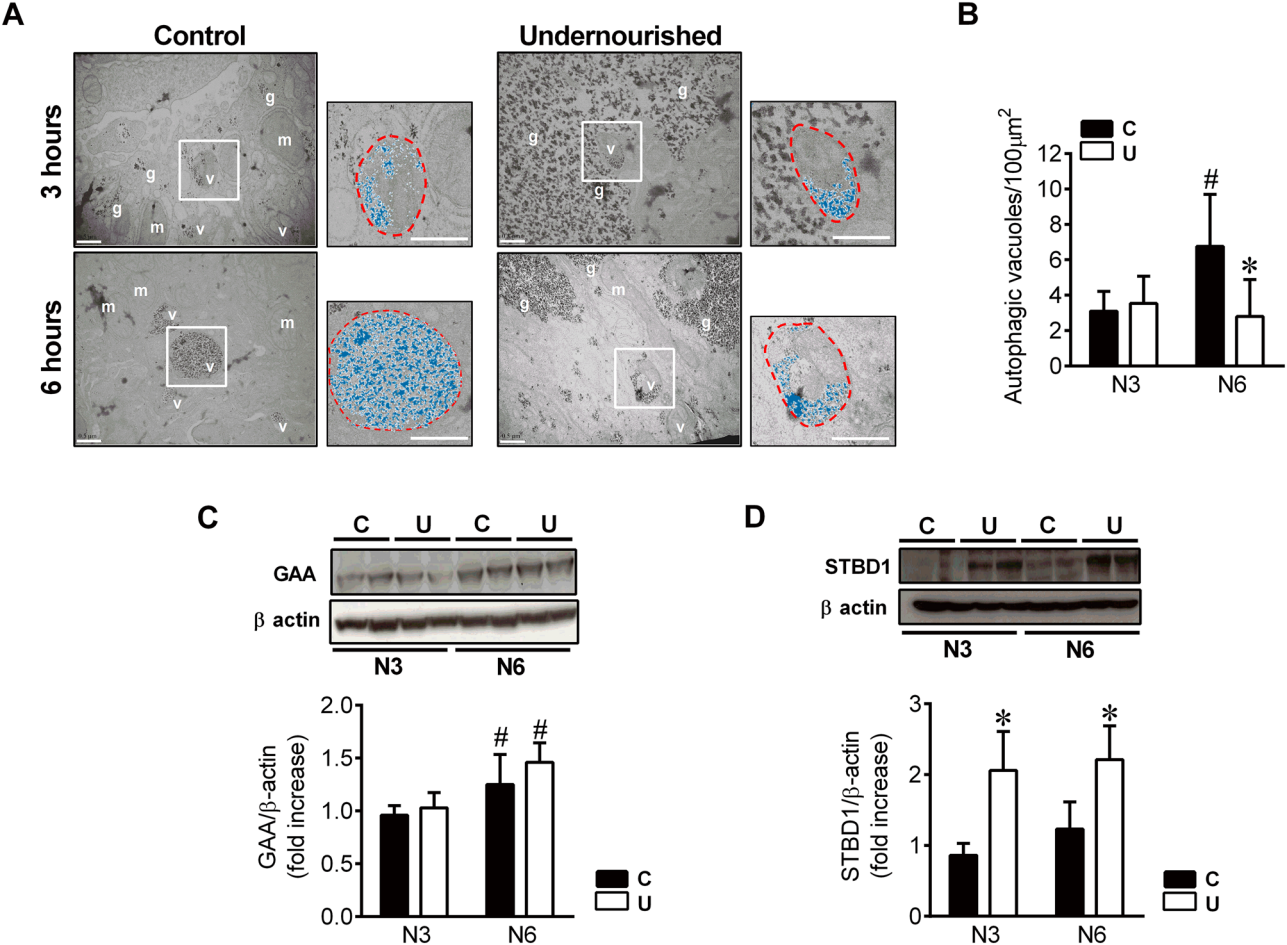
Reduced mobilization of glycogen into autophagic vacuoles in the liver of IUGR neonates. (**A**) Representative electron microscopy images after silver proteinate staining showing autophagosomes and autophagolysosomes in hepatocytes from C and U neonates at N3 and N6. Insets show higher magnification images of autophagic vacuoles with glycogen inside coloured digitally in blue. m: mitochondria, g: glycogen, v: autophagic vacuoles (scale bar, 500 nm). (**B**) Number of autophagic vacuoles per 100μm^2^ of hepatic tissue quantified from 26 micrographs captured along a total cytoplasmic area of 1500μm^2^ for each animal, n = 3–4. All values are mean ± SD. *p < 0.05 U versus C neonates at the same time point. #*p* < 0.05 N6 versus N3 within the same nutritional condition. Representative blots of 3 independent experiments (n = 6) of the protein levels of lysosomal alpha-glucosidase enzyme (GAA) (**C**) and starch binding domain protein 1 (Stbd1) (**D**) in liver homogenates from N3 and N6 neonates. Bars represent densitometric quantification of all blots relative to β-actin protein levels. All values are mean ± SD. *p < 0.05 U versus C neonates at the same time point. #*p* < 0.05 N6 versus N3 within the same nutritional condition.

The analysis of autophagy machinery showed that Beclin-1 protein levels were not altered by IUGR or postnatal fasting (Fig. 5B), while Atg7 levels were significantly reduced in N6U neonates, as compared to N6C animals (*p* = 0.048). In agreement with previous studies^5^, C newborns showed a marked induction of microtubule-associated protein 1B-light chain 3 (LC3B) II conversion between 3 and 6 h, whereas in U animals LC3B I lipidation did not change after 6 h of fasting. To elucidate whether the decrease in LC3B II levels was due to reduced formation rather than increased clearance of autophagosomes, we analysed the levels of another recognized marker of autophagic flux, the sequestome-1 protein (SQSTM1/p62)^23^. The accumulation of p62 protein levels at both 3 and 6 h after birth (Fig. 5B) confirmed the impairment of autophagic flux in U animals. Quantitative TEM of livers from N3C and N6C neonates showed abundant autophagic vesicles containing high amounts of glycogen, particularly at 6 h of life (Fig. 6A, B). In agreement, almost no cytosolic glycogen was observed at this time point. By contrast, the number of autophagic vesicles was clearly reduced in N6U newborns and those observed contained little glycogen inside. However, vast areas of cytosolic glycogen were found in these animals, as previously indicated, together with increased lipid droplet accumulation (Supplementary Fig. S1).

Along with the increase of autophagic activity in C newborns between 3 and 6 h of life, the protein levels of lysosomal alpha-glucosidase enzyme GAA also rose up in this time period, without significant differences when compared to restricted animals (Fig. 6C).

Finally, starch binding domain protein 1 (Stbd1 or genethonin 1), a membrane and glycogen-binding protein, has been shown to be involved in the transfer of abnormal glycogen to lysosomes^24^. However, its role in liver glycogen mobilization during neonatal fasting has not been tested. Although Stbd1 protein levels slightly increased in both C and U neonates in response to fasting, no significant differences were found between 3 and 6 h for any of the two groups (Fig. 6D). Noteworthy, Stbd1 protein content was markedly higher in both N3U (*p* = 0.008) and N6U (*p* = 0.027) as compared to values observed in C animals.

### Glucagon treatment of undernourished neonates failed to prevent hepatic glycogen accumulation

**Figure 7:**
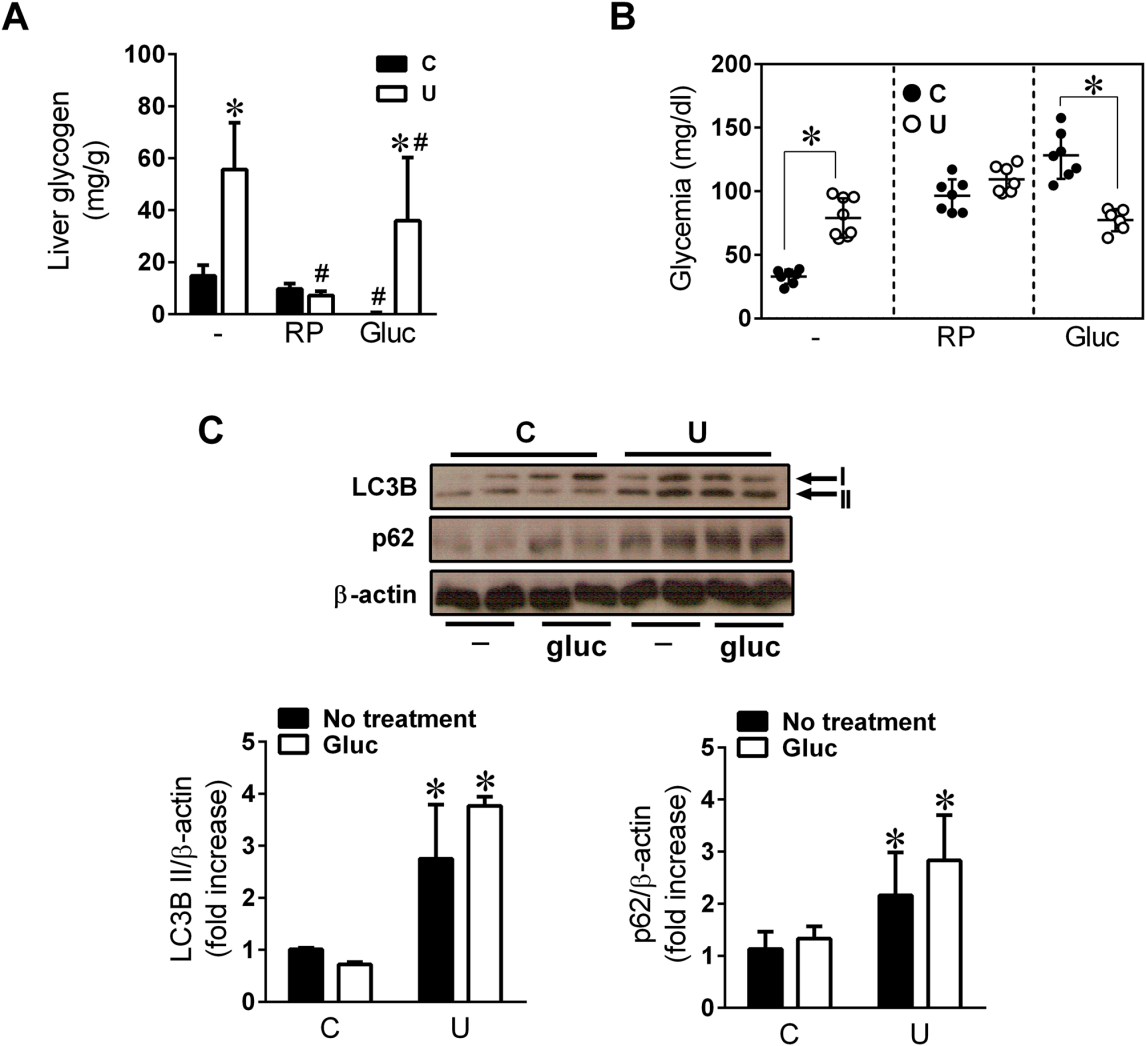
Glucagon treatment of U neonates fails to induce autophagy-dependent mobilization of liver glycogen. C and U neonates were treated or not with a single intraperitoneal dose of rapamycin (RP; 5 mg/kg) or glucagon (GN; 20 mg/kg) at birth and then 3 h-fasted (N3) before measurement of (**A**) liver glycogen content or (**B**) basal glycemia, n = 6–12. All values are mean ± SD. *p < 0.05 U versus C neonates within the same treatment. #*p* < 0.05 RP-treated or GN-treated versus untreated N3 neonates within the same nutritional condition. (**C**) Protein extracts from livers of C and U neonates at N3, RP- or GN-treated, were immunoblotted for autophagy markers LC3B and p62. Graphical bars represent densitometric quantification relative to β-actin protein levels from 4 individual animals per group. All values are mean ± SD. *p < 0.05 U versus C neonates under the same treatment.

To further investigate whether autophagy might play an important role in the observed liver glycogen and lipid accumulation, the neonates were treated at birth with rapamycin (RP, 5 mg/kg BW), a commonly used pharmacological activator of autophagy, or with glucagon (GN, 20 mg/kg BW). Treatment with RP significantly reduced liver glycogen content in N3U + RP animals as compared to non-treated N3U (*p* < 0.0001), to near N3C + RP levels (Fig. 7A). This event was associated to a concomitant increase of glycemia in both RP-treated populations as compared to their corresponding non-treated littermates (*p* < 0.0001, N3C + RP vs. N3C; *p* = 0.0008, N3U + RP vs. N3U) (Fig. 7B). The effect of rapamycin on hepatic glycogen content was due to stimulation of autophagy rather than inhibition of insulin signalling in view of the hyperstimulation of Akt observed in RP-treated neonates (Supplementary Fig. S2). In addition, glucagon treatment also drastically reduced hepatic glycogen in N3C + GN neonates (Fig. 7A), inducing a 3.6-fold increase in glycemia compared with non-treated N3C animals (Fig. 7B). In sharp contrast, liver glycogen content remained significantly high in the liver of N3U + GN neonates (Fig. 7A) and no variation in serum glucose values was observed in response to exogenous administration of glucagon indicating that the lack of glycogen mobilization in U hepatocytes was not only due to the very low levels of circulating glucagon (Fig. 7B).

To confirm that glycogen autophagy was in fact glucagon-dependent, we examined autophagy flux in the liver of N3C and N3U neonates treated with glucagon at birth (Fig. 7C). LC3B II protein levels were reduced 3 h following glucagon treatment in the liver of N3C animals but it remained significantly elevated in N3U neonates. Accumulation of the cargo protein p62 in the liver of N3U + GN animals hence confirmed the lack of autophagy-inducing effect of glucagon in U rats. Taken together, these data support the notion that the loss of glucagon action observed in N3U neonates may be related to the suppressive effect of insulin in autophagy rather than glucagon resistance since inhibition of mTOR with rapamycin was able to induce autophagy even in hyperinsulinemic conditions.

### High-fat diet-induced hepatic lipid accumulation leads to activation of the ER stress marker eIF2α in U adult rats

**Figure 8:**
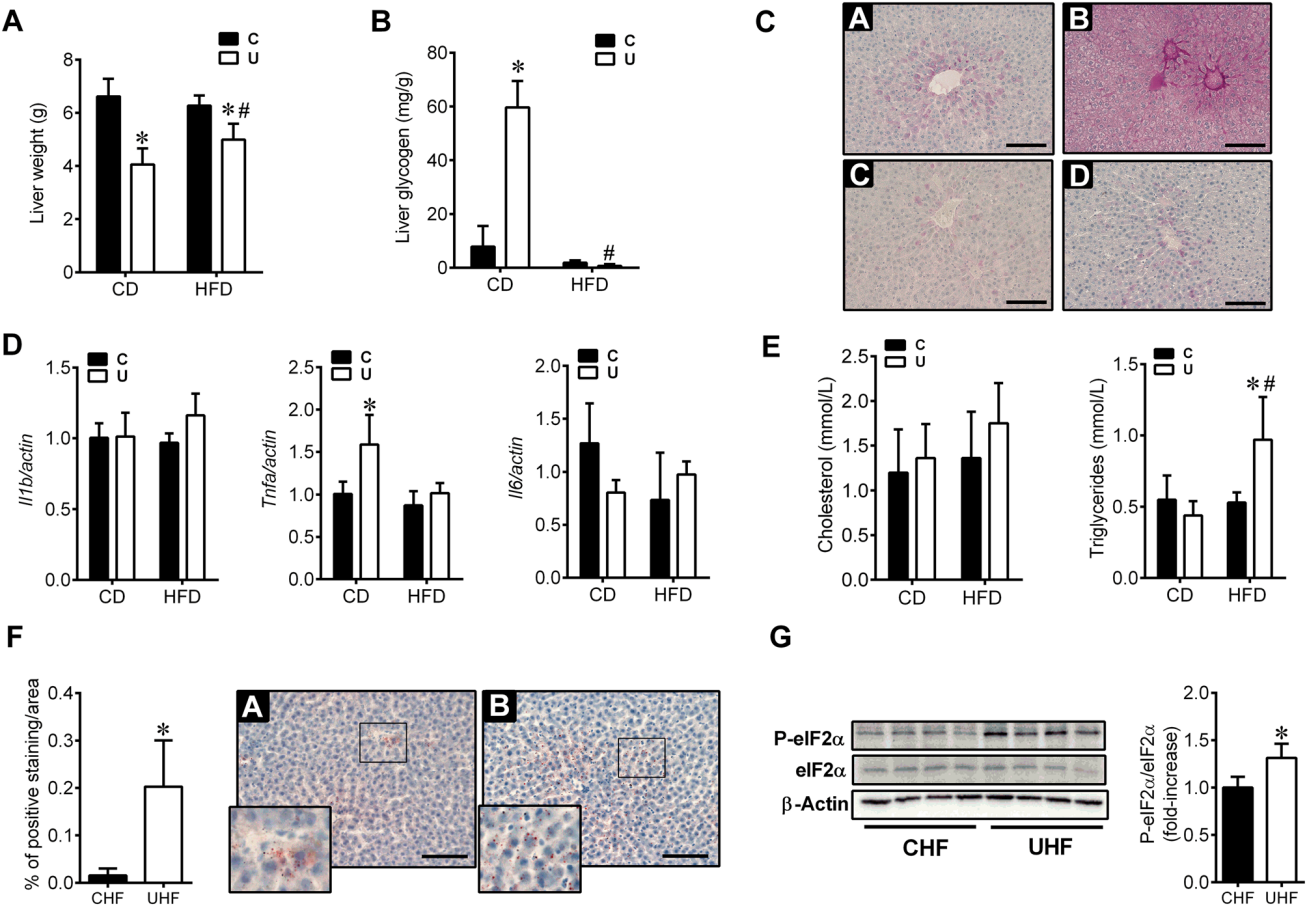
High-fat diet-induced hepatic lipid accumulation leads to activation of the ER stress marker eIF2α in U adult rats. (**A**) Liver weight of control (C) and undernourished (U) adult rats fed with chow-diet (CD) or high-fat diet (HFD) for 22 weeks, n = 10–12. (**B**) Glycogen content in livers (n = 5–6) and (**C**) periodic acid-Schiff (PAS) staining of liver sections from C and U adult rats fed with CD or HFD (n = 2) (scale bar, 100 μm). (**D**) Quantitative RT-PCR analysis of *Il1b*, *Tnfa* and *Il6* expression on adult rat livers of different diet groups normalised for actin (n = 5–6). (E) Cholesterol and triglycerides circulating levels (mmol/L) in C and U rats fed with chow-diet (CD) or high-fat diet (HFD) for 22 weeks (n = 12). (**F**) Representative images of Oil Red O staining and quantification of hepatic-specific lipid content in C and U rats fed with HFD for 22 weeks (CHF and UHF) (n = 3) (scale bar, 100 μm). (**G**) Representative blots of 2 independent experiments (n = 8) of the protein levels of phosphorylated and total eIF2α in liver homogenates from CHF and UHF animals. Bars represent densitometric quantification of all blots relative to β-actin protein levels. All values are mean ± SD. *p < 0.05 within the same type of diet (U vs. C; UHF vs. CHF); # *p* < 0.05 between different diet groups (CHF vs. C; UHF vs. U).

To evaluate to what extent hepatic alterations occurring at birth in U newborns may predispose them to deregulated carbohydrate and lipid metabolism in the adulthood, especially when challenged with a HFD from weaning, we performed biochemical, histological and genomic approaches in the liver of female rats. As shown in Fig. 8A, liver weight increased in food-restricted rats after 22 weeks on HFD (UHF). On the contrary, no liver weight gain was observed in CHF animals as compared to C group. However, the liver weight gain in UHF rats was not enough to overcome the liver weight of C or CHF rats. On the other hand, U rats continued showing significantly high glycogen stores in the liver at adulthood in agreement with their insulin hypersensitivity at this age (Fig. 8B, C)^18,25^. However, 22 weeks on HFD totally prevented glycogen accumulation in the liver of UHF rats suggesting the development of insulin resistance in the liver (Fig. 8B, C). Analysis of hepatic cytokine expression did not reveal differences in the inflammatory profile among nutritional groups, except for *Tnfa* expression in U rats as compared to C animals (Fig. 8D).

We also observed that U rats under HF-feeding developed mild hypertriglyceridemia whereas no effect of diet was seen in C animals either for cholesterol or triglyceride circulating levels (Fig. 8E). Moreover, as shown in Fig. 8F, HFD caused a moderate liver steatosis in both groups of animals (CHF and UHF) with significant higher lipid accumulation in the offspring from U mothers. This event was associated to activation of the endoplasmic reticulum (ER) stress marker eIF2α in UHF rats but not in CHF animals (Fig. 8G).

## Discussion

Inappropriate maternal nutrition during perinatal development negatively impacts on the metabolic programming of the offspring, increasing the risk of developing diseases such as obesity and T2D^20^. This study demonstrates that an impairment in hepatic autophagy compromises physiological glycogen breakdown in U offspring, thereby hampering the metabolic adaptation of IUGR rats to extrauterine life. The mechanism of defective autophagy is likely to be dependent on hormonal factors, since not only sustained hyperinsulinemia and mTORC1-mediated signaling were observed in U neonates, but also the levels of glucagon were decreased at birth.

It is well-established that rat fetal liver accumulates large glycogen stores short before delivery^26^. Herein, we report significantly higher levels of liver glycogen in U rats at birth, probably due to enhanced glycogen synthase activity compared to C animals. In agreement with our data, other authors have also described higher glycogen stores in the liver of fetuses at term^27^ or suckling rats^28,29^ submitted to maternal low-protein diet. Likewise, Li et al.^30^ reported increased liver glycogen in fetuses from globally restricted baboons, indicating that this response is independent of the type of restriction or species studied. As expected, during the first hours of postnatal life, C neonates depleted hepatic glycogen reserves almost entirely, while U newborns still maintained high levels of hepatic glycogen and even displayed an increase during the first 3 h of life. Paradoxically, this situation suggested that anabolic processes would be favored in a situation of strong energy demand. In this regard, it has been reported that the accumulation of glycogen in an animal model of Lafora disease^31^ can cause autophagy deficiency per se, suggesting that the autophagy impairment is likely the consequence and not the cause of glycogen accumulation. However, others have also reported autophagy deficiency in the liver of IUGR newborn piglets, but associated with significantly reduced hepatic glycogen content^32^.

In the very early perinatal period, numerous studies have linked neonatal glycogen autophagy induction with starvation and high circulating levels of glucagon, concomitantly with a fall in plasma insulin^2,33^. Indeed, the drop in insulin appears to be the key event in the induction of autophagy^33^. It is noteworthy that newborns from U mothers showed a marked hyperinsulinemia at birth^34,35^, together with lower plasma glucagon concentrations^36^ compared to the controls. Under these conditions it is difficult to determine the respective importance of each hormone. However, several lines of evidence indicate that high plasma insulin levels at delivery and during the early postnatal period impair hepatic glucose output. On one hand, the crucial role played by hyperinsulinemia in preventing glycogen breakdown is supported by the ability of anti-insulin serum injected at delivery to promote a prompt decrease in liver glycogen concentration, not only in normal newborns, but also in the offspring of diabetic mothers which are characterized by elevated levels of circulating insulin^37^. Moreover, it has been described that insulin plays a dominant role over glucagon in controlling liver autophagy because, even under high plasma glucagon levels, autophagy is only induced when circulating insulin levels drop^33^. Thus, the abnormal high I/G ratio exhibited by U neonates would favor mTORC1 activation and, consequently, the inhibition of autophagy. An interesting finding of our study is that pharmacological activation of autophagy with rapamycin significantly reduced the vast glycogen accumulation in the liver of U newborns to the levels of C animals even under hyperinsulinemic conditions. However, this effect was markedly attenuated in glucagon-treated U rats, pointing out that autophagy machinery is not essentially damaged in these animals. Thus, altered neonatal regulation of hepatic glucose output from glycogen stores is likely to be primarily related to hyperinsulinemia along with blunted counterregulatory glucagon response. These effects, together with the delay in PYGL and PEPCK enzyme induction, might compromise the survival of neonates during fasting. Unexpectedly, U newborns did not develop hypoglycemia during the very early hours of life. In fact, they showed higher blood glucose levels when compared with C animals. Similar results have been observed in fetuses of mothers with gestational hyperglycemia^38^. Due to dual adaptation to food-restriction and pregnancy, U pregnant rats show disturbances in glucose homeostasis characterized by glucose intolerance and impaired in vivo insulin secretion at the end of gestation^35^ similar to that found in mild diabetic mothers^38,39^.

It should be pointed out that there is a marked dissociation between the dysfunction of the mechanisms of autophagosome-derived glucose production or other pathways such as gluconeogenesis, and the blood glucose levels detected in IUGR newborns. These apparently contradictory results are explained, at least in part, by decreased levels of GLUT-4 and -1 in the skeletal muscle of U neonates. Although we have not assessed insulin-dependent translocation of glucose transporters in muscle, it is described that the level of GLUT-4 expression determines the maximal effect of insulin on glucose transport^40^. Decreased rate of glucose uptake would ensure glucose availability mainly for the brain and consequently, neonatal survival. However, this specific programming of insulin resistance might participate at the long-term in the pathophysiology of T2D. Accordingly in humans, impaired regulation of *Glut4* gene expression in skeletal muscle and adipose tissue has been observed in insulin-resistant subjects born with IUGR^41^.

On the other hand, we cannot exclude the possibility that the observed effects in our study are partly associated to an aberrant structure of glycogen from fetal origin similarly to what has been described in Lafora disease^31,42^ where poorly branched, hyperphosphorylated insoluble glycogen-like polymers accumulate in neurons, muscle, heart, liver, and several other tissues. This would render glycogen granules inaccessible to debranching enzyme or glycogen phosphorylase whereas glycogen mobilization does occur in rapamycin-treated animals.

We are aware that the observations regarding the metabolic state of the rats at birth may not be directly extrapolated to other mammals, because of a number of peculiar features of this species, including the almost absence of white adipose tissue, the use of glycogen as primary energy store, and a very high basal metabolic rate. However, both a decrease in glycemia and a shift in circulating insulin and glucagon levels have also been reported in the human newborn^43^. The secretory behavior of the human islet a- and b-cells at birth may thus be relevant to normal extrauterine adaptation, and altered secretion might contribute to abnormal states of glucoregulation, as demonstrated in the infants of diabetic mothers^37,38,44^. In this regard, the herein reported failure of glycogen mobilization seems to be associated with a delay in neonatal maturation of the hepatic mechanisms necessary for endogenous glucose production. The blockage of hepatic autophagy flux would be then a consequence of these metabolic adaptations developed by IUGR newborns to deal with the scarcity of nutrients. However, when challenged with a hypercaloric diet these metabolic adaptations compromise hepatic function favoring the development of liver steatosis, ER stress and ultimately insulin resistance. Similar results have been recently described in the offspring of 70% food-restricted dams under HFD which exhibited modifications of global transcriptome profile and altered lipid metabolism in the liver^45^. Although with limitations, the influence of prenatal adversity on transcriptional regulation of specific genes involved in pathways related to energy production and growth have also been addressed in humans prenatally exposed to famine, for example during the Dutch Hunger Winter at the end of World War II. Using systematic genome-wide approach, Tobi EW and co-workers^46^ showed that epigenetic mechanisms, such as DNA methylation (DNAm) mediates the associations between prenatal famine exposure and later-life adiposity or serum TG levels through the regulation of genes involved in energy metabolism (*PIM3*), b-cell function (*TXNIP*), glycolysis (*PFKFB3*), and adipogenesis (*METTL8*). These findings, which were performed in whole blood, are in agreement with other studies reporting association in DNAm data from metabolic relevant tissues either in humans^47,48^ or in animal models^49^.Although transcriptomic approaches are beyond the scope of our study, the overall physiological meaning of our results are in agreement with the previous data regarding the influence of maternal nutrition in metabolic programming of offspring’s tissues. Therefore, we believe the present study could provide additional insights into the mechanisms of disturbed fuel homeostasis in individuals with prenatal growth restriction and its long-term consequences.

## Methods

### Reagents

Rapamycin (Vetranal), Tween-80, PEG-400, periodic acid, amiloglucosidase, silver proteinate (Protargol), Oil Red O, monoclonal anti-insulin (I2018), monoclonal anti-glucagon (G2654), anti-β-Actin (A5316) and anti-IRS-1(06–248) antibodies were from Sigma-Aldrich (St. Louis, MO, USA). Glucagon (GlucaGen Hypokit) was purchased from Novo Nordisk (Copenhagen, Denmark). Hematoxylin was from Panreac Quimica (Barcelona, Spain). Schiff’s reagent was from VWR Prolabo Chemicals (Barcelona, Spain). Bradford reagent, acrylamide and Immunoblot PVDF membrane were from Bio-Rad (München, Germany). Pierce ECL Western Blotting Substrate was purchased from ThermoFisher Scientific (Waltham, MA, USA). Anti-phospho-GSK3αβ (no. 9331), anti-phospho-GS (Ser641) (3891), anti-GS (no. 3886), anti-phospho-AMPK (Thr172) (no. 25355), anti-AMPK (no. 2532), anti-phospho-mTOR (Ser 2448) (no. 2971), anti-mTOR (no. 2972), anti-Atg7 (no. 2631), anti-LC3 (no. 4108), anti-p62 (SQSTM1) (no. 5114), anti-phospho-eIF2α (Ser51) (no. 9721), anti-eIF2α (no. 9722), anti-phospho-Akt (Ser473) (no. 9271), anti-phospho-Akt (Thr308) (no. 9275), anti-Akt (no. 9272) and anti-phospho-S6 Ribosomal (Ser240/244) (no. 5364) antibodies were from Cell Signaling Technology (Boston, MA, USA). The anti-IRβ (sc-711), anti-GSK3αβ (sc-7291), anti-GR (sc-66912), anti-PYGL (sc-46347), anti-PEPCK (sc-32879), anti-Beclin (sc-11427), anti-LYAG/GAA (sc-67358), anti-GLUT 1 ((A4) sc-377228) and anti-GLUT 4 ((IF8) sc-53566) antibodies were from Santa Cruz Biotechnology (Santa Cruz, CA, USA). Anti-Stbd1 antibody (11842-1-AP) was purchased from Protein Tech Group (Chicago, USA).

### Animals and diets

Two weeks after mating Wistar pregnant rats were randomly separated into two groups. The control (C) group (n = 6) was fed ad libitum with a standard chow diet (A04, SAFE, Villemoisson-sur-Orge, France) and the undernourished (U) group (n = 6) was 65% food-restricted from the last third of gestation, as previously described^18^. In order to study liver glycogen metabolism during the first hours of life we practised a caesarean to dams at embryonic day 21.5, just before the normal time of birth, and sequential changes in the same metabolic parameters were quantified during the first 3 h and 6 h of the neonatal period. Newborns were maintained in a humidified thermostatic-controlled chamber at 37ºC and 70% relative humidity, and remained unfed for the whole of the present study. Adequate blood collection in newborns, via an incision across the axillary vessels, necessitated exsanguination; hence, each time period is represented by different animals. Individuals from a given litter were employed for different time periods, such that several litters were studied longitudinally, for any given parameter. Because all measurements could not be performed on a single animal, at least two determinations of hormones or substrates were made to overlap with other groups, ensuring that for a given time point, all individuals represented a homogenous metabolic state. A total of four different groups of animals were then analyzed in the present study: neonates from C mothers at 3 h (N3C) and 6 h (N6C), and neonates from U mothers at 3 h (N3U) and 6 h (N6U). No difference between groups was observed in the mortality of newborns during the first hours of life.

For long-term studies, C or U fetuses were let to naturally born and maintained with their respective mothers in the same feeding pattern along lactation. The litters were standardized to 8 pups per nursing dam to minimize effects of litter size on postnatal growth. At weaning, rat pups from both groups were randomized to continue on their diet (C and U, respectively) or to switch to a high-fat diet (control high-fat [CHF] and undernourished high-fat [UHF]), which received ad libitum for 22 weeks (HFD; 4.7 kcal/g; 45% fat, 35% carbohydrates and 20% protein; D12451, Research Diets). All studies were conducted according to the principles and procedures outlined by the Federation of European Laboratory Animal Science Associations (FELASA) and approved by the Animal Ethics Committee of the Complutense University of the Autonomous Community of Madrid (PROEX 349/15), Spain.

### Pharmacological treatments

When necessary, C and U neonates were treated at birth with glucagon or rapamycin, two activators of autophagy. Glucagon-treated (GN) animals were given at birth an intraperitoneal injection of 0.1 ml solution (20 mg/kg) of GlucaGen or saline and then euthanized after 3 h of fasting, whereas rapamycin-treated (RP) animals were injected at birth with 0.1 ml of Vetranal (5 mg/kg in 5% Tween-80/ 5% PEG-400) and then euthanized at the same time point. In all cases, the liver was rapidly removed, weighed, and either frozen at − 70 °C for subsequent glycogen or protein extraction, or immersed in fixation solution for histological analyses.

### Analytical analysis

Serum glucose levels were determined by glucose oxidase method (Biosystems, Barcelona, Spain). Serum insulin and glucagon were determined by specific rat radioimmunoassay (RIA, Merck Millipore, MA, USA). Sensitivity of 0.1 ng/ml and of 20 pg/ml was achieved with overnight equilibrium using 100 μl serum samples, respectively. The coefficient of variation within and between assays were 10% for insulin and 4.0 and 7.3%, respectively for glucagon.

### In vitro insulin and glucagon secretion

Islets were isolated and cultured as previously described^36^. Insulin and glucagon secretion was measured by RIA after exposure of islets to different glucose concentrations.

### Liver glycogen content

Glycogen was extracted from neonatal livers in 30% KOH saturated with Na_2_SO_4_, precipitated in 95% ethanol and incubated with amyloglucosidase to hydrolyze the glycogen^17^. Glucose resulting from enzymatic hydrolysis was quantified by the glucose oxidase method. Glycogen content was finally expressed as milligrams of glucose per gram wet liver tissue.

### Enzymatic activities

The activities of phosphoenolpyruvate carboxykinase (PEPCK)^50^ and glycogen phosphorylase (PYGL)^51^ were assayed by standard spectrophotometric procedures.

### Histology assessments and transmission electron microscopy in liver

Liver samples were fixed overnight in 4% paraformaldehyde (PFA) in 0.1 M phosphate buffer pH 7.4 and routinely paraffin embedded. Serial sections (5 μm) were mounted on glass slides, hydrated and stained with hematoxylin or with periodic acid-Schiff (PAS) reagent to detect glycogen. For estimation of hepatic lipid content, liver samples were included in optimum cutting temperature (OCT) compound, frozen in liquid nitrogen and serially sectioned in a cryostat to perform Oil Red o staining using standard techniques. Immunohistochemistry in paraffin-embedded pancreatic sections for detection of insulin and glucagon-positive cells was performed as previously described^36^. Images of all stained sections were acquired using a digital camera (XCD-U100CR; Sony, Tokyo, Japan) attached to a light microscope (Eclipse 80i; Nikon, Tokyo, Japan). The color considered as positive staining for the same protein was manually selected and the value corresponding to the sum of all the stained areas was obtained with Histolab software (Microvision Instruments). The results were expressed as the percentage of stained area with respect to the total area analyzed in each sample.

For transmission electron microscopy (TEM), tissues were obtained and immediately fixed in 2.5% glutaraldehyde and 4% PFA in 0.1 M phosphate buffer pH 7.4 at room temperature. After post-fixation in 2% OsO_4_ (osmium tetroxide), blocks were processed for embedding in Epon 812 resin. Thin sections were obtained, stained with uranyl acetate and lead citrate, and examined by TEM in a JEOL EX 1200 electron microscope. The number of autophagy vesicles was quantified and expressed by 100 µm^2^ of hepatic tissue. The mean values were obtained from 26 micrographs and from a total cytoplasmic area of 1500 µm^2^. Silver proteinate staining was used to demonstrate glycogen presence through a Schiff reaction with polysaccharide hydroxyl groups exposed by periodic acid oxidation^52^.

### Preparation of protein extracts and western blot

Sample tissues were homogenized with a Polytron in ice-cold lysis buffer (17), composed of 50 mM HEPES (pH 7.4), 150 mM NaCl, 1 mM MgCl2, 10 mM sodium pyrophosphate, 10 mM sodium fluoride, 2 mM EDTA, 10% glycerol, 1% Nonidet P-40, 2 mM phenylmethylsulfonyl fluoride, 2 mM benzamidine, 10 μM leupeptin, 10 μg/ml aprotinin, and 2 mM sodium orthovanadate. After protein content determination with Bradford reagent, total protein was boiled in Laemmli sample buffer^53^ and submitted to 8–15% SDS-PAGE. Proteins were transferred to Inmunoblot PVDF membrane, and after blocking with 3% BSA or 5% non-fat dry milk, incubated overnight with several antibodies as indicated. Immunoreactive bands were visualized using and enhanced chemiluminiscence reagent (Amersham Life Science, Little Chalfont, Buckinghamshire, UK). Normalization of western blot was ensured by reprobing the blots with mouse anti-rat β-actin and densitometric analysis of the bands was performed using the Image J software (NIH, Bethesda, MD, USA).

### RNA extraction and quantitative RT-PCR

Total RNA was extracted from frozen livers using TRIzol Reagent (Invitrogen, ThermoFisher Scientific, MA, USA) and reverse transcribed using a high-capacity cDNA reverse transcription kit (Applied Biosystems, ThermoFisher Scientific). Real-time quantitative PCR analyses were performed using TaqMan probes (Applied Biosystems) to determine the relative abundance of *Il1b* (Rn00580432_m1), *Il6* (Rn01410330_m1) and *Tnfa* (Rn99999017_m1). The comparative threshold cycle method was used to calculate the relative expression. The target gene values were normalized to the expression of the endogenous reference (*Actb*; Rn00667869_m1).

### Statistical analyses

Results for all measurements are expressed as the mean ± SD. Significance was tested by 2-tailed Student’s t test for comparing two groups. For comparisons of more than two groups, the statistical significance of differences was analysed by one-way or two-way ANOVA followed by post hoc Tukey’s test. Significance was considered at *p* < 0.05. GraphPad Prism 7 software (San Diego, CA) was used to analyse the results.

## Supplementary information

Supplementary information.
