# Supplementary material for: Defective liver glycogen autophagy related to hyperinsulinemia in intrauterine growth-restricted newborn wistar rats
Source: Sci Rep. 2020 Oct 19;10:17651. doi: 10.1038/s41598-020-74702-9 (PMC7573689; doi:10.1038/s41598-020-74702-9)

DEFECTIVE LIVER GLYCOGEN AUTOPHAGY RELATED TO HYPERINSULINEMIA IN INTRAUTERINE GROWTH-RESTRICTED NEWBORN WISTAR RATS

Juan de Toro-Martín[1], Tamara Fernández-Marcelo[2], Águeda González-Rodríguez[3,4], Fernando Escrivá[2,5], Ángela M. Valverde[2,6], Carmen Álvarez[2,5]*, Elisa Fernández-Millán[2,5]*.

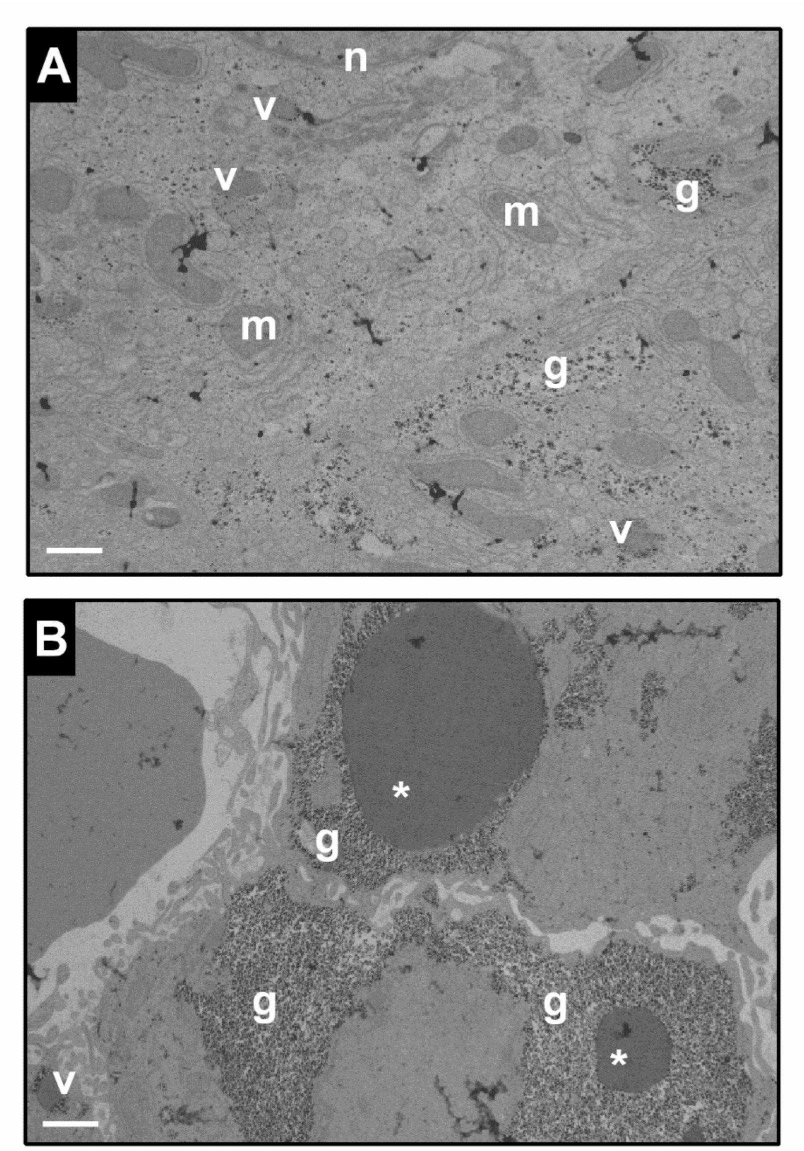

Supplementary Figure S1. **IUGR neonates show increased hepatic lipid content.** Ultrastructural analysis of the livers of control (C) and undernourished (U) neonates by electron microscopy shows increased lipid droplet accumulation in the liver of U animals. Representative image of C (A) and U (B) liver section. N: nucleus; m: mitochondria; g: glycogen; v: autophagic vacuole; asterisk: lipid droplet (scale bar: 1 μm).

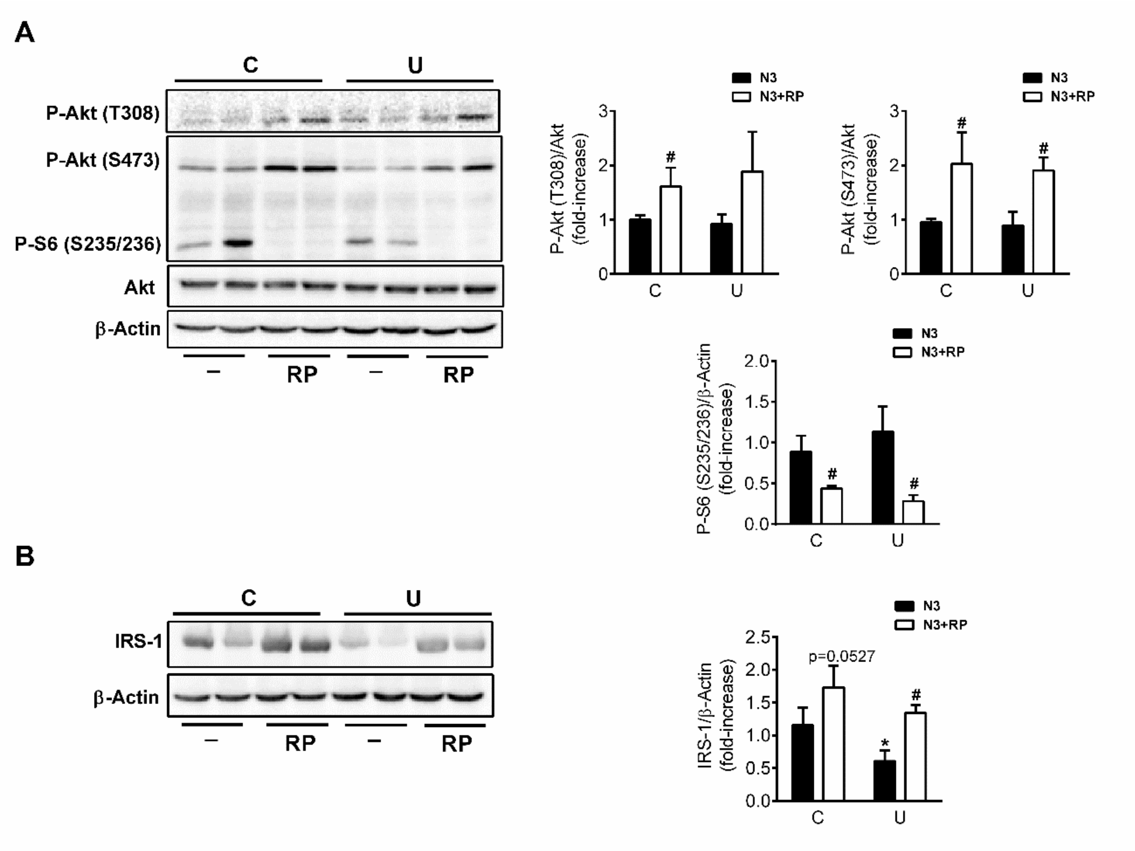

Supplementary Figure S2. Short-term treatment with rapamycin increased IRS-1 protein content and Akt-mediated insulin signalling in the liver of C and U neonates. C and U neonates were treated or not with a single intraperitoneal dose of rapamycin (RP; 5 mg/kg) at birth and then 3 h-fasted (N3). Hepatic protein extracts were immunoblotted using specific antibodies against phospho-Akt (T308), phospho-Akt (S473), Akt, phospho-S6 ribosomal (S235/236) (**A**) and IRS-1 (**B**). Graphical bars represent densitometric quantification relative to β-actin protein levels from 4 individual animals per group. All values are mean ± SD. *p < 0.05 U vs. C neonates under the same treatment. #p < 0.05 N3+RP vs. N3 for the same nutritional group of neonates.

Supplementary Figure S3. Expanded blots shown in main Figure 3

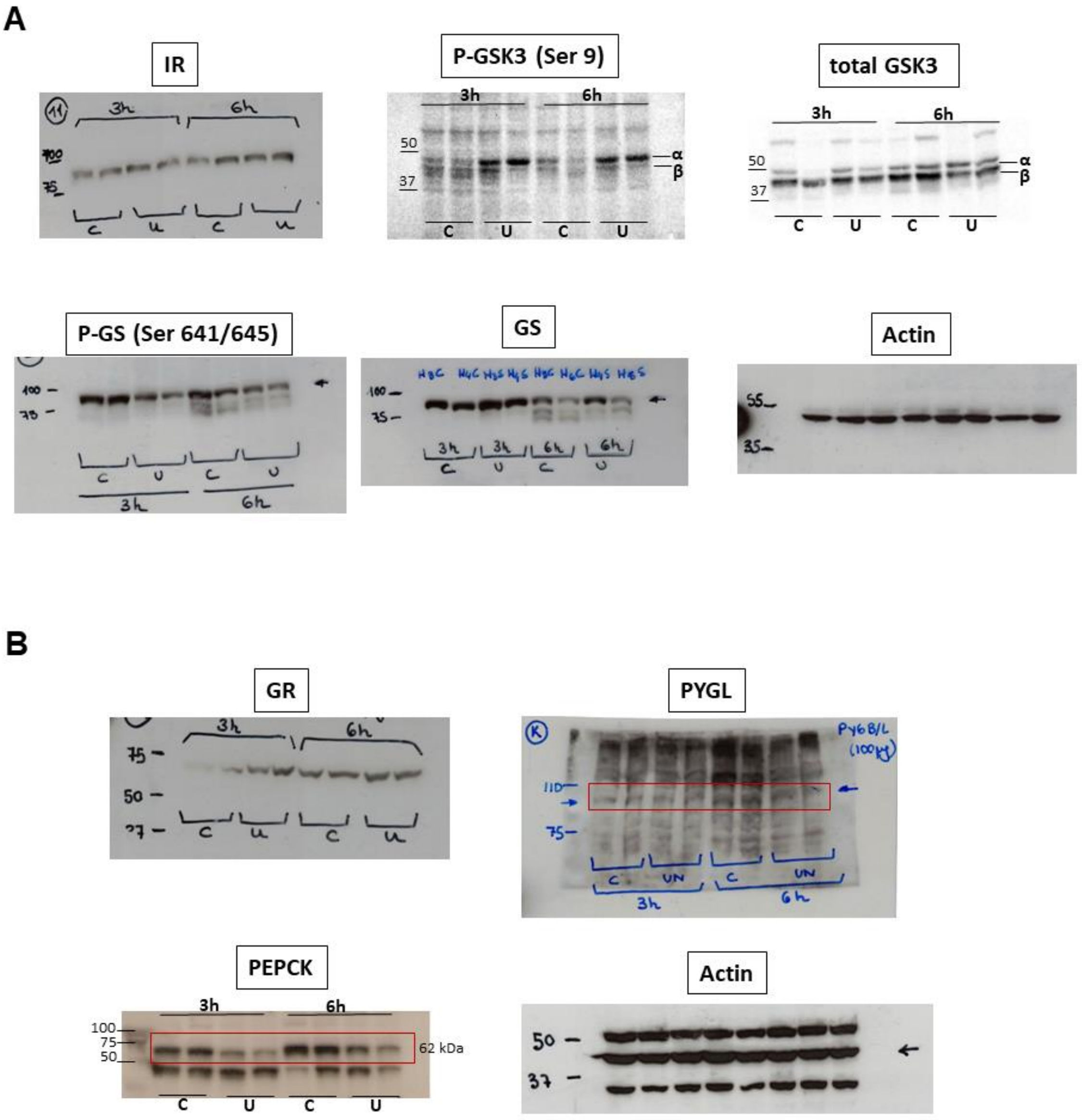

Supplementary Figure S4. Expanded blots shown in main Figure 4

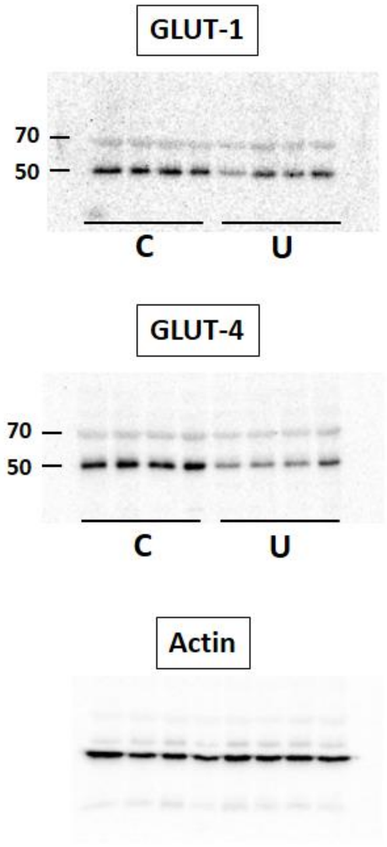

Supplementary Figure S5. Expanded blots shown in main Figure 5

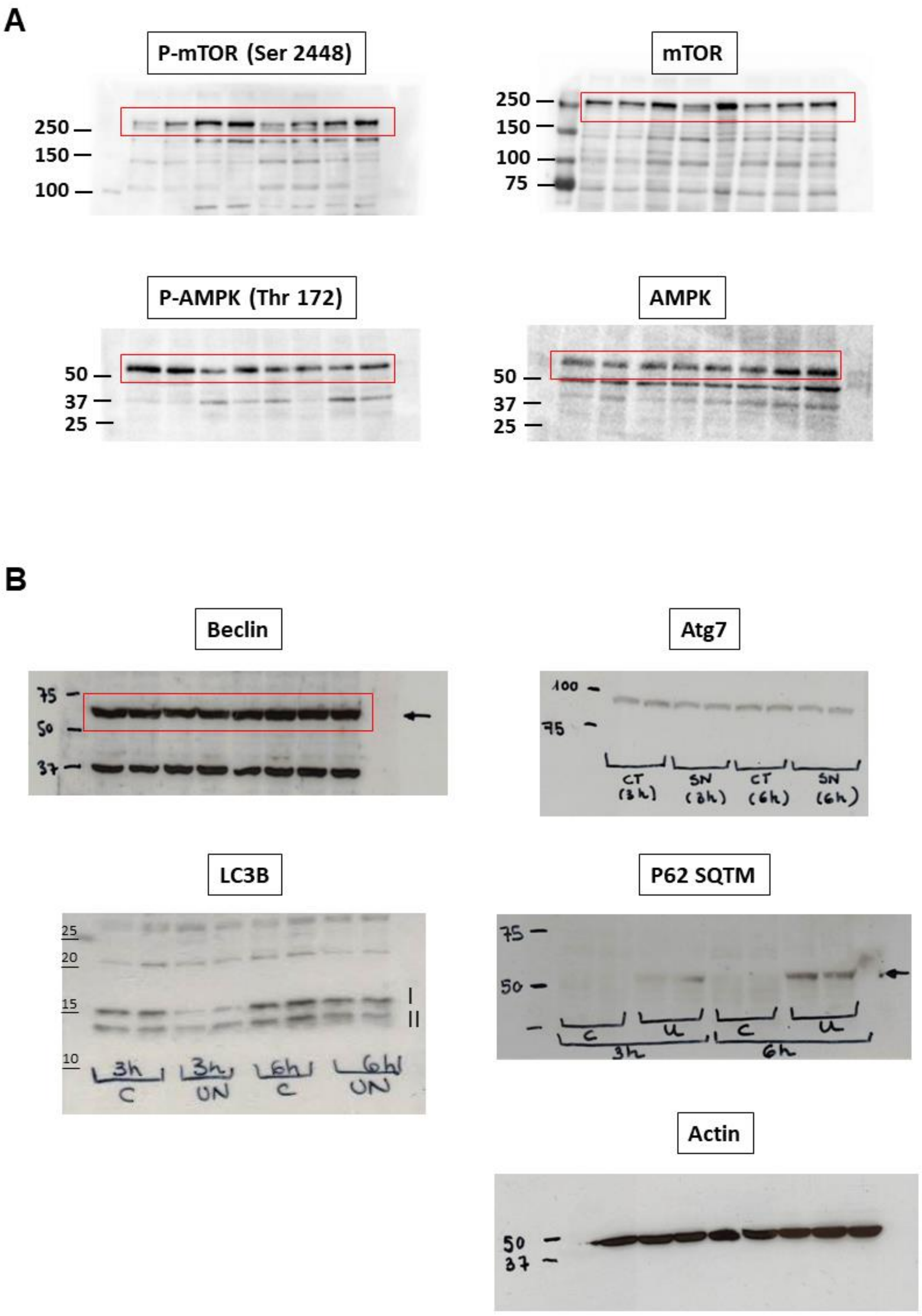

Supplementary Figure S6. Expanded blots shown in main Figure 6

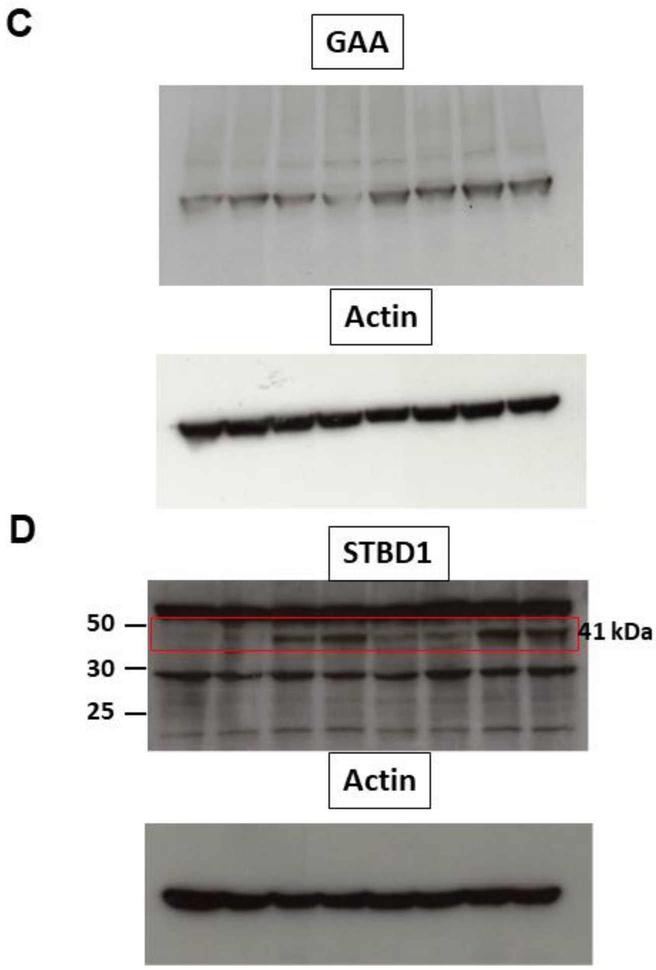

Supplementary Figure S7. Expanded blots shown in main Figure 7

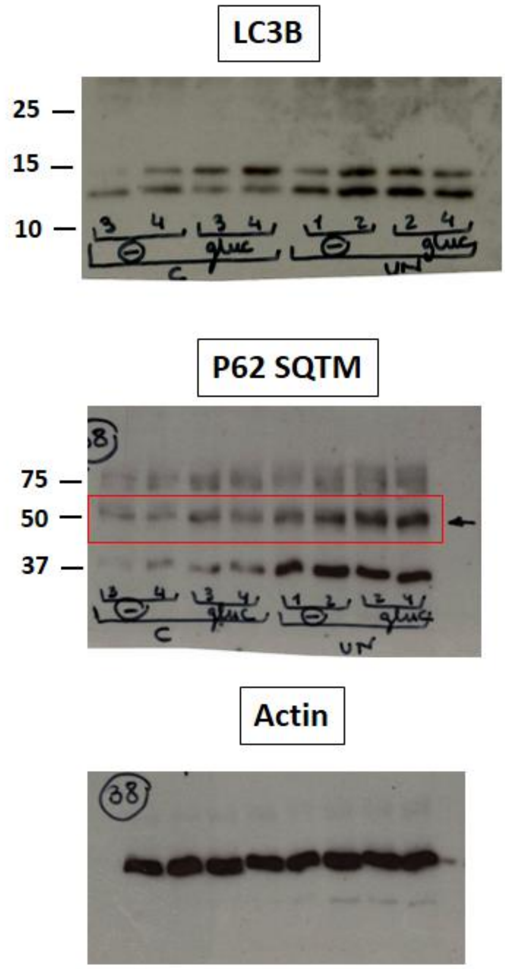

Supplementary Figure S8. Expanded blots shown in main Figure 8

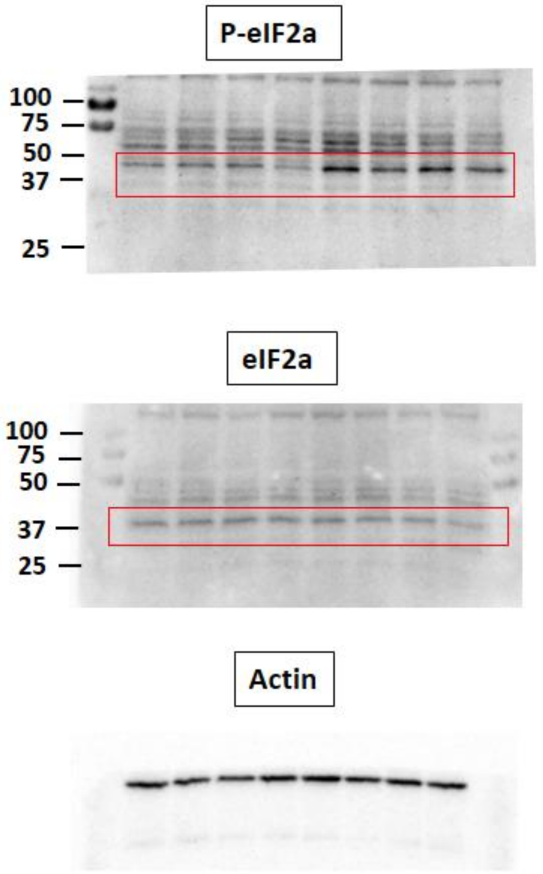

Supplementary Figure S9. Expanded blots shown in supplementary Figure S2

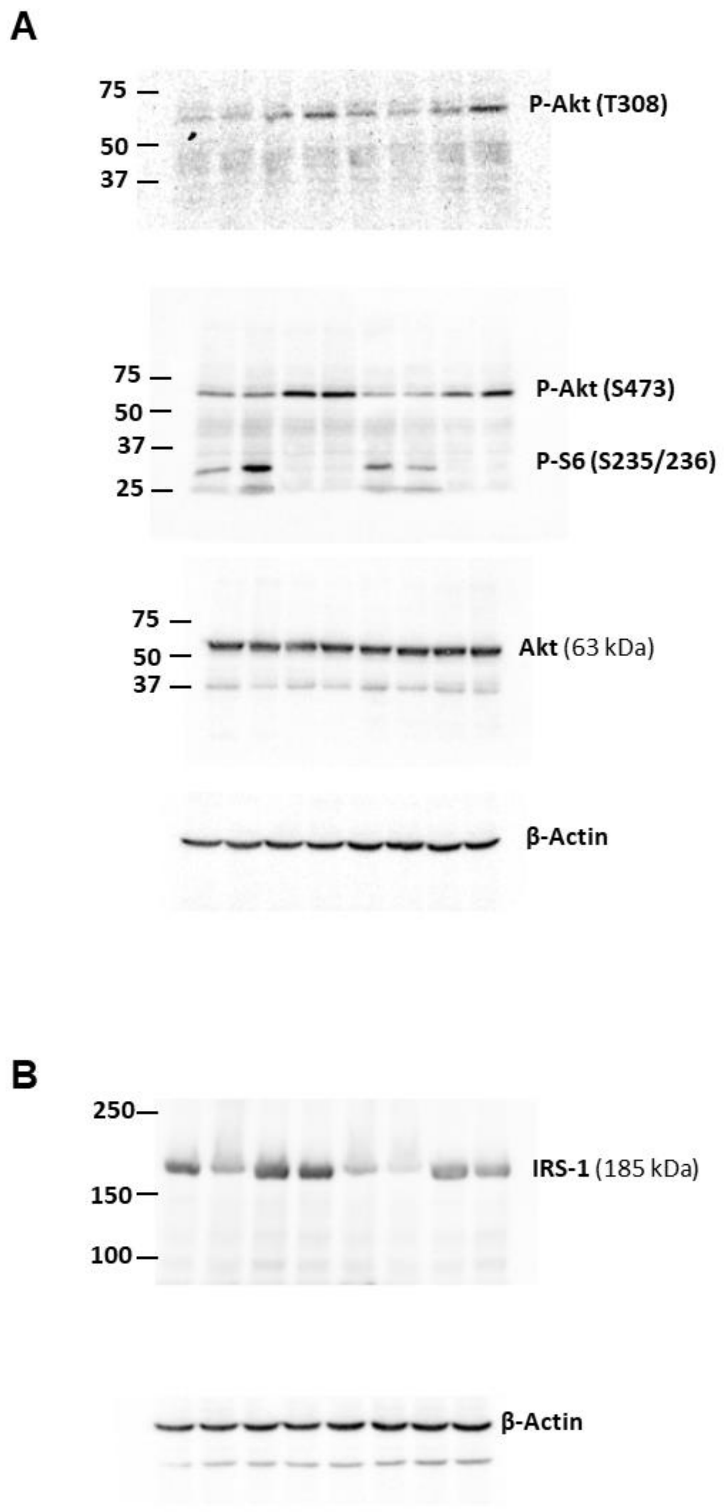

Supplement: Supplementary file 1 — Supplementary information. [file 41598_2020_74702_MOESM1_ESM.pdf]
